# VPS37A Activates the Autophagy-Lysosomal Pathway for TNFR1 Degradation and Induces NF-**κ**B-Regulated Cell Death under Metabolic Stress in Colorectal Cancer

**DOI:** 10.32604/or.2025.065739

**Published:** 2025-07-18

**Authors:** Chuncheng Liu, Xiaohan Liu, Ziqi Li, Yanruoxue Wei, Bangdong Liu, Peng Zhu, Yukun Liu, Ran Zhao

**Affiliations:** 1 Cheeloo College of Medicine, Shandong University, Jinan, 250012, China; 2 Department of Pathology, Affiliated Hospital of Jining Medical University, Jining Medical University, Jining, 272029, China

**Keywords:** VPS37A (VPS37A subunit of ESCRT-I), tumor necrosis factor receptor 1 (TNFR1), nuclear factor kappa-B (NF-κB) signaling, metabolic stress, lysosomal degradation, colorectal cancer (CRC)

## Abstract

**Background:**

VPS37A (VPS37A subunit of ESCRT-I), a component of the ESCRT-I (endosomal sorting complex required for transport I) complex, mediates vesicular trafficking through sorting endocytic ubiquitinated cargos into multivesicular bodies (MVBs). Although accumulating evidence indicates that VPS37A deficiency occurs in numerous malignancies and exerts tumor-suppressive effects during cancer progression, its functional significance in colorectal cancer (CRC) pathogenesis remains poorly characterized. Therefore, this study aims to further investigate the functional and molecular mechanisms by which VPS37A downregulation contributes to malignant biological phenotypes in CRC, with a specific focus on how its dysregulation affects cell death pathways.

**Methods:**

Multi-omics analysis of TCGA, GEO, and CPTAC cohorts identified VPS37A as a downregulated tumor suppressor gene in CRC. The prognostic relevance of VPS37A was validated in two clinical cohorts (Cohorts 1 and 2) using immunohistochemistry. Functional assays in VPS37A-overexpressing CRC cells and xenografts assessed proliferation, cell cycle progression, and stress-induced cell death. RNA sequencing, nuclear factor kappa-B (NF-κB) luciferase reporter assays, and lysosomal inhibition experiments elucidated the mechanisms underlying tumor necrosis factor receptor 1 (TNFR1) degradation.

**Results:**

VPS37A is significantly downregulated in advanced-stage CRC and independently predicts poor survival. Functionally, VPS37A overexpression suppresses proliferation and induces G2/M arrest *in vitro*, while reducing xenograft growth. Under metabolic stress (glucose deprivation/galactose adaptation), VPS37A triggers cell death via apoptosis, necroptosis, and ferroptosis. Mechanistically, VPS37A redirects TNFR1 to lysosomal degradation, suppressing NF-κB nuclear translocation and transcriptional activity.

**Conclusion:**

VPS37A deficiency drives CRC progression by sustaining TNFR1/NF-κB signaling under metabolic stress. Restoring VPS37A activity promotes TNFR1 degradation, offering a therapeutic strategy to counteract NF-κB-mediated treatment resistance in CRC.

## 1 Introduction

Colorectal cancer (CRC) remains the second-leading cause of global cancer mortality, with metastatic 5-year survival rates below 15% despite molecularly targeted therapies [1–3]. Therapeutic resistance in CRC increasingly associates with rewired cell death pathways—including apoptotic desensitization [4], necroptosis bypass [5,6], ferroptosis resistance [7,8], pyroptosis inhibition [9], and cytoprotective nucleophagy [10,11]—underscoring the need to delineate context-specific survival mechanisms.

Central to malignancy adaptation, the nuclear factor kappa-B (NF-κB) pathway integrates stress signaling [12,13], nutrient sensing [14–16], death evasion [17], and immune modulation [18,19] to drive treatment resistance [20]. Notably, stress-inducible Tumor Necrosis Factor Receptor 1 (TNFR1) internalization has emerged as a druggable regulator sustaining NF-κB activation in tumors [21,22], with its subcellular trafficking (lysosomal degradation vs. plasma membrane recycling) representing a key therapeutic target [23,24].

VPS37A (VPS37A subunit of ESCRT-I), a core component of the endosomal sorting complex required for transport I (ESCRT-I) protein complex [25], orchestrates ubiquitin-dependent cargo sorting and membrane remodeling critical for multivesicular body (MVB) biogenesis and lysosomal degradation [26,27]. Its context-dependent roles in cancer biology reveal striking tissue specificity. In hepatocellular carcinoma (HCC), VPS37A downregulation activates Signal Transducer and Activator of Transcription 3 (STAT3)-driven metastasis [28], while its deficiency impairs autophagy-lysosomal degradation, compromising mitochondrial quality control and exacerbating genomic instability in early tumorigenesis [29]. Notably, the pathophysiological contribution of VPS37A to colorectal cancer progression is incompletely characterized and remains to be elucidated.

This study aims to systematically investigate the molecular mechanisms underlying VPS37A downregulation in driving malignant phenotypic manifestations in CRC, with a dedicated emphasis on delineating how its dysregulation modulates cell death signaling cascades through molecular intermediaries. Here, the findings position VPS37A deficiency as both a prognostic biomarker and an actionable metabolic liability in CRC, establishing TNFR1/NF-κB pathway inhibition as a tailored therapeutic strategy for CRC patients.

## 2 Materials and Methods

### 2.1 Clinical Samples and Ethical Approval

This study analyzed two independent CRC cohorts from the Affiliated Hospital of Jining Medical University (Shandong, China). Cohort 1 (*n* = 60) included 30 CRC specimens and their paired non-cancerous tissues (Supplementary Table S1), while Cohort 2 (*n* = 156) comprised surgically resected CRC patients with complete follow-up data (Table 1). All participants provided written informed consent, with protocols approved by the Ethics Committee of Affiliated Hospital of Jining Medical University (Approval No. 2023-11-B002). Overall survival (OS) was calculated from diagnosis to death or last follow-up. All specimens were pathologically confirmed using the American Joint Committee on Cancer (AJCC) 8th edition staging criteria.

**Table 1:** Correlation between the expression level of VPS37A and the clinical pathological characteristics of CRC (N = 156)

Characteristic	VPS37A expression (IHC intensity (%))		**χ** ^**2**^	*p*-value
	High (*n* = 73)	Low (*n* = 83)		
**Age (years)**			7.921	0.005**
>60 (*n* = 87)	32 (36.78)	55 (63.22)		
≤60 (*n* = 69)	41 (59.42)	28 (40.58)		
**Sex**			1.731	0.188
Female (*n* = 62)	25 (40.32)	37 (59.68)		
Male (*n* = 94)	48 (51.06)	46 (48.94)		
**Location**			0.601	0.438
Colon (*n* = 57)	29 (50.88)	28 (49.12)		
Rectum (*n* = 99)	44 (44.44)	55 (55.56)		
**Tumor size**			0.834	0.361
>4 cm (*n* = 73)	37 (50.68)	36 (49.32)		
≤4 cm (*n* = 83)	36 (43.37)	47 (56.63)		
**LNM status**			2.066	0.151
Positive (*n* = 65)	26 (40.00)	39 (60.00)		
Negative (*n* = 91)	47 (51.65)	44 (48.35)		
**Differentiation grade**			/	0.230^a^
Well (*n* = 6)	5 (83.33)	1 (16.67)		
Moderate (*n* = 97)	44 (45.36)	53 (54.64)		
Poor (*n* = 53)	24 (45.28)	29 (54.72)		
**TNM stage**			5.194	0.023*
Stages I + II (*n* = 81)	45 (55.56)	36 (44.44)		
Stages III + IV (*n* = 75)	28 (37.33)	47 (62.67)		

Note: ^a^Fisher’s exact test; *p < 0.05; **p < 0.01.

### 2.2 Immunohistochemistry (IHC) Staining and Quantitative Scoring

Tissue specimens were fixed in formalin, paraffin-embedded, and sectioned into 4-μm slices. Immunostaining was performed using anti-VPS37A antibody (1:200; Santa Cruz, Dallas, TX, USA) following the protocol described previously [30]. Two pathologists independently scored staining intensity at 200× magnification using a semi-quantitative scale: 0 (negative), 1 (weak), 2 (moderate), 3 (strong). Samples with scores ≥2 were defined as high-expression.

### 2.3 Cell Culture, Stable Cell Line, and Metabolic Stress Modeling

Human CRC cells SW620 and DLD-1 were purchased from the Typical Cultures Preservation Center of the Chinese Academy of Sciences (Shanghai, China). All cells were authenticated by short tandem repeat (STR) profiling and confirmed mycoplasma-free through testing. Cells were cultured in RPMI-1640 (Gibco, Waltham, MA, USA) with 10% FBS and 1% penicillin/streptomycin at 37°C/5% CO_2_. For stable VPS37A overexpression, the full-length coding sequence (CDS; NM_152415.3) was cloned into GV341 lentiviral vectors (GeneChem, Shanghai, China). Cells were transduced with VPS37A-expressing or control lentiviruses, selected with 2 μg/mL puromycin (Invitrogen, Carlsbad, CA, USA) for 72 h, and validated by qRT-PCR (primers listed in Supplementary Table S2) and Western blotting (antibodies listed in Supplementary Table S3). Metabolic stress was induced by either Galactose adaptation (10 mM galactose, 2 mM glutamine, 1 mM pyruvate in glucose-free RPMI-1640, 36 h) or acute glucose deprivation (GD; 0.5 mM glucose in RPMI-1640, 24 h).

### 2.4 Cell Proliferation and Colony Formation Assay

Cell proliferation was assessed by seeding cells in 96-well plates (1000 cells/well) and measuring viability with CCK-8 (GLPBIO, Montclair, CA, USA). OD450 values were recorded at the indicated time points using a BioTek Synergy LX Multimode Reader (Agilent BioTek, Winooski, VT, USA). For colony formation, 500 cells/well were plated in 6-well plates in triplicate. After 14 days, colonies were fixed with 4% paraformaldehyde, stained with 0.1% Giemsa (Beyotime, Shanghai, China), and quantified manually. Three independent experiments were performed.

### 2.5 Cell Cycle and Cell Death Analysis

Cell cycle and apoptosis analyses were performed as previously described [30]. Briefly, cells were fixed in 70% ethanol (−20°C, ≥2 h), treated with RNase A (100 μg/mL; Beyotime, Shanghai, China), and stained with PI (25 μg/mL; Beyotime, Shanghai, China) for 30 min at 37°C in darkness. DNA content was analyzed via flow cytometry (CytoFLEX, Beckman Coulter, Brea, CA, USA). For apoptosis quantification, cells were stained with Annexin V-PE/7-AAD (BD Biosciences, San Jose, CA, USA) or PI (50 μg/mL; Beyotime, Shanghai, China) per manufacturer’s protocol. Early apoptotic and late apoptotic populations or necrotic cells were discriminated, respectively. At least three independent experiments were performed.

### 2.6 RNA Extraction and Quantitative Real-Time PCR (qRT-PCR)

Total RNA was isolated from VPS37A-overexpressing cells or control cells with TRIzol (Invitrogen, Carlsbad, CA, USA) following the manufacturer’s guidelines. cDNA synthesis used RevertAid First Strand Kit (Thermo, Waltham, MA, USA). qRT-PCR was performed using gene-specific primers purchased from Sangon Biotech (Shanghai, China) (Supplementary Table S2) and TB Green Premix Ex Taq II (Takara Bio, Shiga, Japan) on a CFX96 system (Bio-Rad, Hercules, CA, USA). Relative mRNA levels were calculated by the 2^−ΔΔCt^ method, normalized to β-actin, with triplicate replicates per sample.

### 2.7 Western Blotting Analysis

Cells were lysed on ice with RIPA buffer (Beyotime, Shanghai, China) containing Roche phosphatase/protease inhibitors. Total protein concentration was determined using a BCA Protein Assay Kit (Beyotime, Shanghai, China) according to the manufacturer’s protocol. Total protein (20–60 μg per lane) was denatured at 95°C in 5× SDS-PAGE Sample Loading Buffer (Beyotime, Shanghai, China), and loaded onto 10–15% SDS-PAGE gels. Proteins were transferred to PVDF membranes (Merck Millipore, Burlington, MA, USA). Membranes were blocked with 5% non-fat dry milk (Beyotime, Shanghai, China) in TBST for 1 h at room temperature. Membranes were incubated with primary antibodies (Supplementary Table S3) diluted in blocking buffer overnight at 4°C. Membranes were washed 3× with TBST (10 min per wash) and incubated with secondary antibodies in blocking buffer for 1 h at room temperature. Protein bands were visualized using Immobilon™ HRP Substrate (Merck Millipore, Burlington, MA, USA). Signals were captured with a Tanon 4600SF imaging system (Tanon, Shanghai, China).

To assess cell death, we also performed Western blotting to detect the following markers: autophagy markers (p62 and LC3), necroptosis markers (MLKL and p-MLKL), ferroptosis marker (GPX4), and apoptosis markers (PARP and cleaved PARP). The overexpression level of VPS37A, as well as its effect on the expression of core protein markers, was assessed by western blotting. In addition, cells were subjected to GD for 6, 8, or 10 h and subsequently harvested for Western blot analysis. Under these conditions, five parallel experimental groups were established: (1) GD; (2) GD with MG132 (5 μM; GLPBIO, Montclair, CA, USA); (3) GD with Rapamycin (100 nM; GLPBIO, Montclair, CA, USA); (4) GD with Chloroquine (25 μM; GLPBIO, Montclair, CA, USA); and (5) GD with CA-5F (10 μM; GLPBIO, Montclair, CA, USA).

### 2.8 Immunofluorescence (IF)

For p65 staining, cells were treated with 100 ng/mL TNF-α (Sino Biological, Beijing, China) for 0.5 h prior to IF staining, and this TNF-*α*-treated group under normal glucose conditions was included as a positive control, while untreated cells maintained in normal glucose-containing medium served as a negative control. IF was performed as previously described [30]. Briefly, cells on glass coverslips were fixed with 4% PFA (15 min, room temperature), permeabilized with 0.3% Triton X-100 (5 min), and blocked with 5% goat serum/TBS (15 min). Primary antibodies (anti-TNFR1, 1:100; sc-8436, Santa Cruz, Dallas, TX, USA; anti-p65, 1:100, #8242, CST, Danvers, MA, USA) were incubated overnight at 4°C, followed by Alexa Fluor 488-conjugated secondary antibody (1:1000; A-21202, Invitrogen, Carlsbad, CA, USA) incubation (1 h, 37°C, dark). Nuclei were counterstained with DAPI (Beyotime, Shanghai, China). Images were acquired using an Olympus BX63 microscope (Olympus, Tokyo, Japan).

### 2.9 Detection of NF-**κ**B Transcriptional Activity

To detect NF-κB transcriptional activity, dual-luciferase reporter assays were performed as described previously [31]. Briefly, VPS37A-overexpressing SW620 and DLD-1 cells or control cells were seeded in 24-well plates at a density of 1 × 10^5^ cells/well and cultured overnight. Cells were then co-transfected with pNFκB-luc (Beyotime, Shanghai, China) and pRL-TK (Promega, Madison, WI, USA) via Lipofectamine 3000 (Invitrogen, Carlsbad, CA, USA). After 24–36 h treatment, firefly/Renilla luciferase activities were quantified using a Dual-Luciferase Kit (Promega, Madison, WI, USA) according to the manufacturer’s protocol.

### 2.10 RNA Sequencing and Bioinformatics

RNA sequencing analysis was performed on SW620/VPS37A and control cells. Total RNA was extracted using TRIzol (Invitrogen, Carlsbad, CA, USA), followed by library preparation and paired-end sequencing (150 bp) on a NovaSeq 6000 platform (Illumina, San Diego, CA, USA). Differentially expressed genes (DEGs) were identified through ‘DESeq2’ (1.40.2) analysis (|log_2_ fold change| > 1, adjusted *p* < 0.05). Data visualization included heatmaps generated by the ‘pheatmap’ package (1.0.12) and volcano plots created with ‘ggplot2’ (3.4.4). The raw sequence data reported in this paper have been deposited in the Genome Sequence Archive in the National Genomics Data Center, China National Center for Bioinformation/Beijing Institute of Genomics, Chinese Academy of Sciences (GSA-Human: HRA010749), which are publicly accessible at https://ngdc.cncb.ac.cn/gsa-human (accessed on 19 March 2025).

For functional annotation, Gene Ontology (GO) biological process terms (Supplementary Table S4) were enriched using ‘clusterProfiler’ (4.15.1) (*p* < 0.05), while pathway analysis integrated Gene Set Enrichment Analysis (GSEA) (the DEGs were listed in Supplementary Table S5) and Gene Set Variation Analysis (GSVA) with Hallmark and KEGG gene sets in Molecular Signatures Database (MSigDB) (https://www.gsea-msigdb.org/gsea/index.jsp (accessed on 19 March 2025)). Transcription factors were predicted via TRRUST v2.0. Pan-cancer expression patterns of VPS37A were analyzed through TIMER2.0 (http://timer.cistrome.org/) based on The Cancer Genome Atlas (TCGA) RNA-seq data (The annotation of cancer types can be found in Supplementary Table S6).

In CRC validation, mRNA expression profiles from GSE39582, GSE71187, and GSE87211 datasets were analyzed via the BEST platform (https://rookieutopia.com/app_direct/BEST/ (accessed on 19 March 2025)), paired colon tissue data were extracted from GSE44076, and proteomic alterations were examined via UALCAN (https://ualcan.path.uab.edu/analysis-prot.html (accessed on 19 March 2025)) using the Clinical Proteomic Tumor Analysis Consortium (CPTAC) colon cancer cohort. Survival correlations were assessed through PanCanSurvPlot (http://www.PanCanSurvPlot.com/) for TCGA data and KM-Plotter (https://kmplot.com) for GSE87211.

### 2.11 In Vivo Xenograft Model

Female BALB/c nude mice were purchased from Jinan Pengyue Laboratory Animal Breeding Company (Jinan, China). A total volume of 100 μL (containing 5 × 10^6^ cells) of VPS37A-stable overexpression SW620 (SW620/VPS37A) cells or control (SW620/Ctrl) cells was subcutaneously inoculated into the right flanks of 6-week-old female BALB/c nude mice (*n* = 5 per group). Tumor growth was monitored every 4 days by measuring the nodules with calipers. Tumor volumes were calculated using the formula described in our previous study [30], and growth curves were plotted accordingly. All mice were euthanized 24 days post-inoculation, and tumors were harvested for final weight measurement. All animal experiments were conducted in accordance with the guidelines and approval of the Ethics Committee of Affiliated Hospital of Jining Medical University (No. 2023-11-B002). The study is reported in accordance with the ARRIVE essential guidelines (https://arriveguidelines.org).

### 2.12 Statistical Analysis

Data represent mean ± SD. Two-tailed Student’s *t*-test for comparisons, chi-square/Fisher’s exact tests for clinicopathological correlations, and log-rank test for survival differences. Cox regression models (uni-/multivariate) assessed VPS37A’s prognostic significance in overall survival (OS). Statistical significance was set at *p* < 0.05. Graphical representations were created with GraphPad Prism v10.

## 3 Result

### 3.1 VPS37A Is Significantly Downregulated in Human CRC Tissues

**Figure 1:**
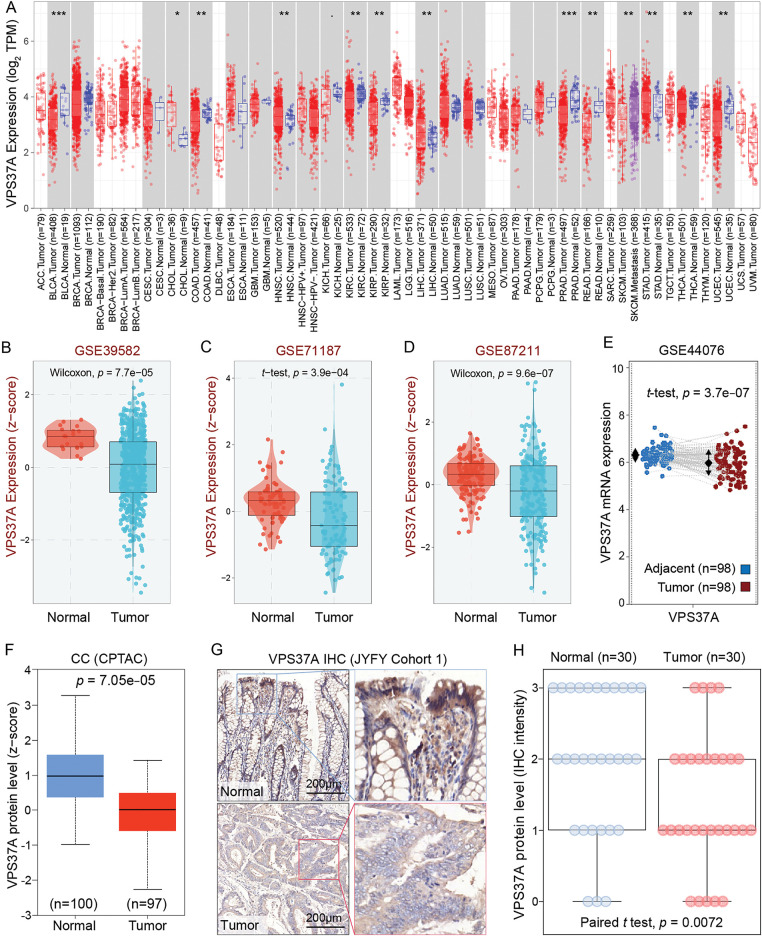
VPS37A is significantly downregulated in CRC. (**A**) Pan-cancer mRNA expression analysis of VPS37A from the TIMER2.0 database. (**B**–**D**) VPS37A mRNA levels in CRC tissues across three independent GEO datasets. (**E**) Paired tumor-normal mRNA expression of VPS37A in COAD from GEO dataset. (**F**) VPS37A protein expression in CRC tissues from CPTAC. Representative IHC staining (**G**) and quantitative analysis (**H**) of VPS37A expression in 30 CRC and 30 paired adjacent normal tissues (Cohort 1; scale bar: 200 μm). *p < 0.05; **p < 0.01; ***p < 0.001

To elucidate the role of VPS37A in CRC, we conducted a comprehensive multi-omics analysis. While ubiquitously expressed in normal tissues, VPS37A showed significant downregulation in numerous malignancies, with particularly pronounced suppression in colorectal (COAD) and rectal adenocarcinomas (READ) (Fig. 1A). Cross-validation using GEO cohorts (GSE39582, *p* = 7.7e−05; GSE71187, *p* = 3.9e−04; GSE87211, *p* = 9.6e−07) (Fig. 1B−D), paired transcriptomes (GSE44076, tumor vs. normal, *p* = 3.7e−07) (Fig. 1E) and CPTAC proteomics (*p* = 7.05e−05) (Fig. 1F) consistently demonstrated VPS37A depletion at both mRNA and protein levels. Clinically, IHC analysis of CRC specimens (Cohort 1, *n* = 60, as listed in Supplementary Table S1) revealed predominant cytoplasmic localization with significantly attenuated tumor staining intensity (*p* = 0.0072; Fig. 1G,H). These multi-omics findings establish VPS37A as a consistently downregulated tumor suppressor in CRC, suggesting its loss may represent an early carcinogenic event.

### 3.2 VPS37A Downregulation Serves as an Independent Predictor of Unfavorable OS in CRC Patients

**Figure 2:**
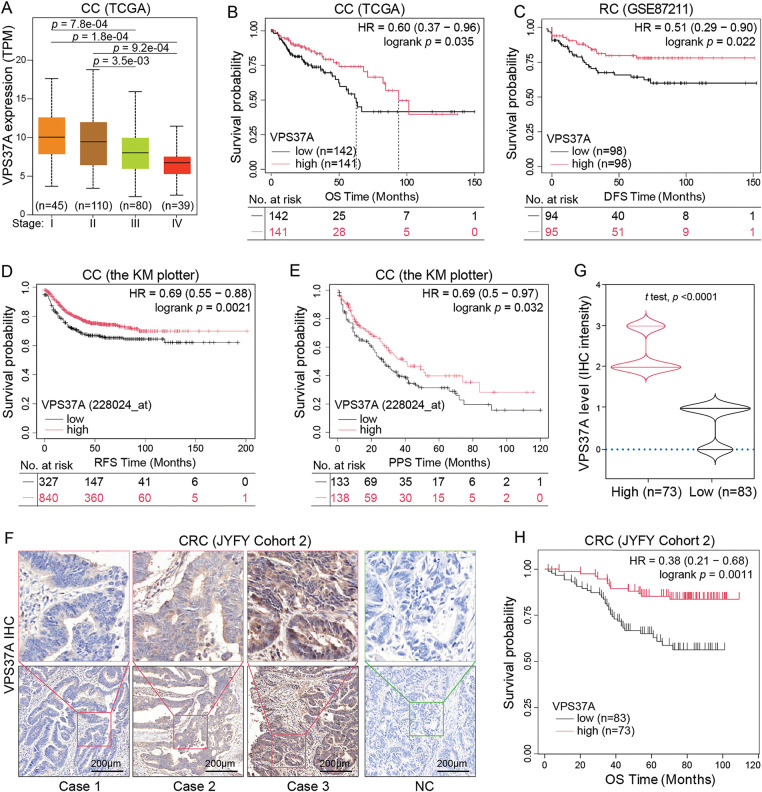
Association between VPS37A expression, clinicopathological features, and survival outcomes in CRC. (**A**) Correlation between VPS37A mRNA levels and tumor stage in CRC (UALCAN database). Kaplan-Meier survival curves of OS in CC (**B**) and disease-free survival (DFS) in RC (**C**) stratified by high/low VPS37A expression (GEO datasets). CC, colon cancer; RC, rectal cancer. Kaplan-Meier analysis of RFS (**D**) and PPS (**E**) in CC from the Kaplan-Meier Plotter database. RFS, recurrence-free survival; PPS, post-progression survival. Representative IHC staining (**F**) and violin plot (**G**) of VPS37A protein expression in CRC tissues (Cohort 2, *n* = 156; high: *n* = 73, low: *n* = 83). (**H**) Kaplan-Meier analysis of OS in CRC patients stratified by VPS37A IHC expression (Cohort 2; log-rank *p* = 0.0011)

To assess the clinical relevance of VPS37A deficiency, we conducted integrated multi-cohort analyses. Analysis of TCGA-COAD data demonstrated a progressive downregulation of VPS37A expression from early-stage to advanced-stage tumors (Fig. 2A). Survival analysis demonstrated reduced overall survival (OS) in low-VPS37A COAD patients (*p* = 0.035; Fig. 2B) and worse disease-free survival (DFS) in READ (*p* = 0.022; Fig. 2C). Cross-validation via the KM Plotter confirmed VPS37A deficiency as a predictor of adverse relapse-free survival (RFS, *p* = 0.0021; Fig. 2D) and post-progression survival (PPS, *p* = 0.032; Fig. 2E).

In the validation cohort (Cohort 2, *n* = 156), IHC quantification stratified 53.2% (83/156) of tumors as VPS37A-low (Fig. 2F,G; Table 1). VPS37A-low status correlated with advanced TNM stages (Stage III/IV: 62.67% vs. 44.44%, *p* = 0.023; Table 1) and predicted poor OS (*p* = 0.0011; Fig. 2H). Multivariate Cox regression identified VPS37A deficiency as an independent prognostic factor (HR = 2.746, 95% CI: 1.390–5.785, *p* = 0.0051) (Table 2). These findings position VPS37A as a robust prognostic biomarker for CRC risk stratification.

**Table 2:** Univariate and multivariate cox regression analysis of the predictors for OS in patients with CRC

Characteristic	Univariate analysis		Multivariate analysis	
	HR (95% CI)	*p*-value	HR (95% CI)	*p*-value
**VPS37A expression**	3.154 (1.667~6.388)	0.0007***	2.746 (1.390~5.785)	0.0051**
Low vs. High				
**Age (year)**	1.944 (1.055~3.770)	0.0391*	1.810 (0.959~3.587)	0.0755
>60 vs. ≤60				
**Sex**	0.939 (0.522~1.720)	0.8350	1.703 (0.907~3.270)	0.1014
Male vs. Female				
**Tumor location**	0.817 (0.454~1.497)	0.5030	0.609 (0.323~1.168)	0.1285
Rectum vs. Colon				
**Tumor size (cm)**	0.922 (0.509~1.658)	0.7863	0.730 (0.393~1.347)	0.3150
>4 vs. ≤4				
**LNM status**	0.691 (0.362~1.265)	0.2437	0.117 (0.048~0.291)	<0.0001****
Positive vs. Negative				
**Differentiation grade**	1.317 (0.706~2.384)	0.3713	1.406 (0.736~2.617)	0.2889
Poor vs. Well-Moderate				
**TNM stage**	1.462 (0.813~2.665)	0.2065	7.399 (2.984~17.490)	<0.0001****
III + IV vs. I + II				

Note: *p < 0.05; **p < 0.01; ***p < 0.001; ****p < 0.0001.

### 3.3 VPS37A Inhibits Cell Proliferation and Tumor Growth of CRC In Vitro and In Vivo

**Figure 3:**
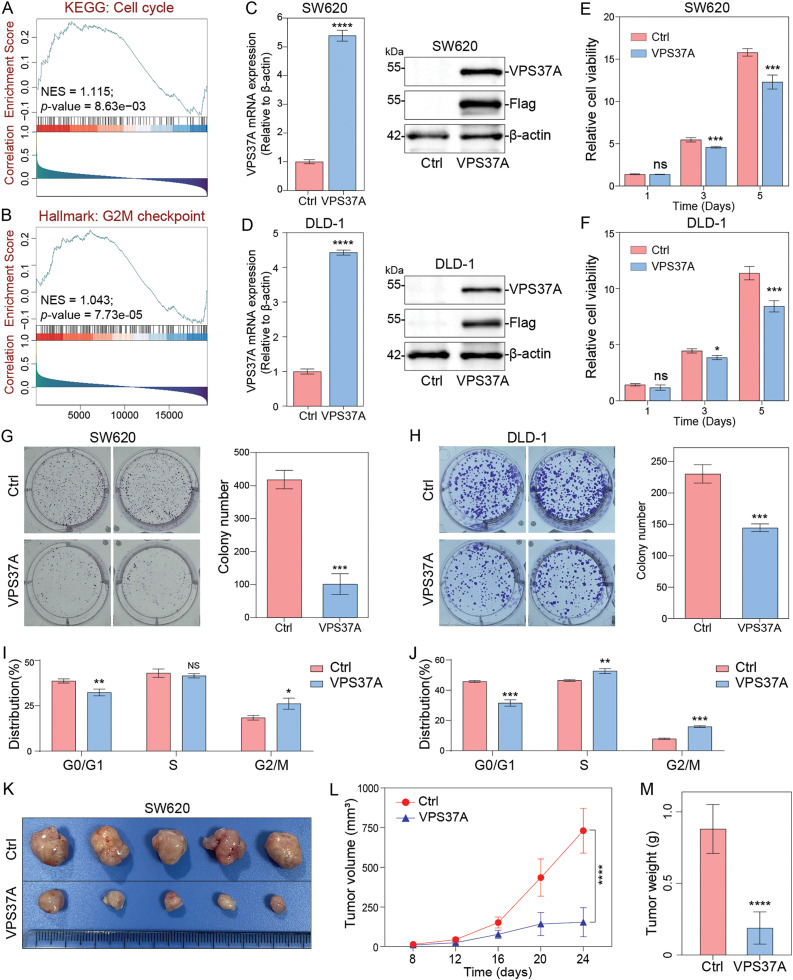
The effects of VPS37A overexpression on proliferation and tumor growth *in vitro* and *in vivo*. GSEA of TCGA CRC datasets showing enrichment of (**A**) the KEGG_Cell cycle pathway and (**B**) Hallmark_G2M checkpoint pathway in VPS37A-overexpressing CRC cells (NES: normalized enrichment score; FDR < 0.05). Validation of VPS37A overexpression efficiency in (**C**) SW620 and (**D**) DLD-1 cells by qRT-PCR (left) and Western blotting (right; β-actin as loading control). Cell viability of (**E**) SW620 and (**F**) DLD-1 cells overexpressing VPS37A assessed by CCK-8 assays (mean ± SD). Colony formation ability of (**G**) SW620 and (**H**) DLD-1 cells after VPS37A overexpression (crystal violet staining; *n* = 3 independent experiments). Flow cytometry analysis of cell cycle distribution in (**I**) SW620 and (**J**) DLD-1 cells overexpressing VPS37A (PI staining; *n* = 3). (**K**) Representative xenograft tumors from nude mice subcutaneously injected with SW620 cells (control vs. VPS37A-overexpressing), (**L**) Tumor volume curves over 24 days, (**M**) Final tumor weights (mean ± SD, *n* = 5 mice per group). ns, no significant; *p < 0.05; **p < 0.01; ***p < 0.001; ****p < 0.0001

To investigate the biological role of VPS37A, we performed GSEA on TCGA CRC datasets. The results showed significant associations between VPS37A expression and the KEGG Cell Cycle pathway (Normalized Enrichment Score (NES) = 1.115, *p* = 8.63e−03) and Hallmark G2/M Checkpoint pathway (NES = 1.043, *p* = 7.73e−05) (Fig. 3A,B), suggesting its potential role in cell cycle regulation during CRC pathogenesis.

To validate these findings, we generated stable VPS37A-overexpressing CRC cells (SW620/VPS37A and DLD-1/VPS37A) and control cells (SW620/Ctrl and DLD-1/Ctrl), confirmed by qRT-PCR and Western blotting (Fig. 3C,D). CCK-8 assays demonstrated that VPS37A overexpression significantly inhibited proliferation in both cell lines (Fig. 3E,F). Colony formation assays revealed even stronger suppression, with VPS37A-overexpressing cells showing fewer colonies than controls (Fig. 3G,H). These results confirm VPS37A’s tumor-suppressive function in CRC.

Mechanistically, flow cytometry analysis showed VPS37A overexpression induced G2/M phase arrest in both cell lines (Fig. 3I,J and Supplementary Fig. S1). *In vivo* subcutaneous xenograft models (*n* = 5 per group) further validated these findings. VPS37A overexpression suppressed tumor growth (*p* < 0.0001) and reduced final tumor weight (*p* < 0.0001) compared to controls (Fig. 3K–M).

Collectively, our data establish VPS37A as a key tumor suppressor in CRC, where its loss promotes oncogenesis through dysregulated cell cycle progression and uncontrolled proliferation.

### 3.4 VPS37A Promotes CRC Cell Death under Metabolic Stress

**Figure 4:**
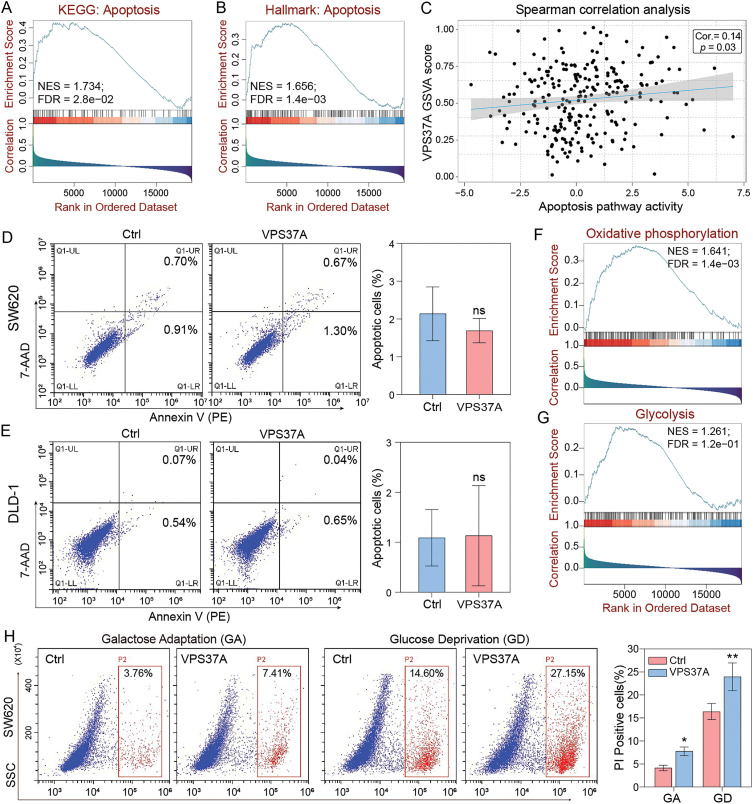

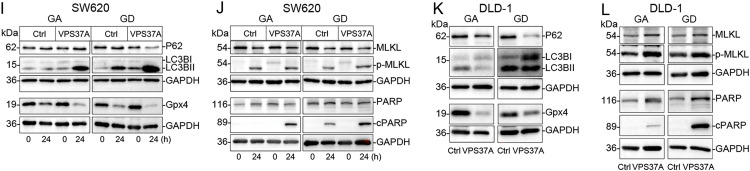
VPS37A induces cell death under energy metabolic stress in CRC. GSEA analyses of (**A**) KEGG apoptosis and (**B**) Hallmark apoptosis pathways. (**C**) Spearman correlation of VPS37A GSVA and apoptosis activity. (**D**,**E**) The effect of VPS37A overexpression on CRC cell apoptosis, as determined by flow cytometry under standard culture conditions. (**F**,**G**) GSEA analysis of TCGA RNA-seq data for metabolic pathways. (**H**) Flow cytometry analysis of cell death using PI staining in SW620 cells under metabolic stress conditions. (**I**–**L**) Western blotting analyses of apoptosis, necroptosis, autophagy, and ferroptosis markers in SW620 and DLD-1 cells under the indicated stress conditions. GA, Galactose Adaptation; GD, Glucose Deprivation. NS, no significant; *p < 0.05; **p < 0.01

Having established VPS37A’s tumor-suppressive role in CRC, we investigated its potential involvement in cell death regulation. GSEA of TCGA CRC data showed significant enrichment of apoptosis pathways (KEGG_APOPTOSIS: NES = 1.734, FDR = 2.8e−02; HALLMARK_APOPTOSIS: NES = 1.656, FDR = 1.4e−03) associated with VPS37A expression (Fig. 4A,B). GSVA further confirmed this correlation (Spearman r = 0.14, *p* = 0.03; Fig. 4C). However, Annexin V-PE/7-AAD flow cytometry revealed no significant apoptosis induction in VPS37A-overexpressing SW620 (*p* > 0.05) or DLD−1 cells (*p* > 0.05) compared to controls (Fig. 4D,E), suggesting context-dependent regulatory mechanisms.

Given emerging evidence of VPS37A’s metabolic regulatory functions, we explored its activity under metabolic stress. Bioinformatics analysis linked VPS37A to oxidative phosphorylation (NES = 1.641, FDR = 1.4e−03) and glycolysis (NES = 1.261, FDR = 1.2e−01) (Fig. 4F,G). We then tested two metabolic stress models: Galactose adaptation (GA) to enhance mitochondrial dependency and Glucose deprivation (GD) to restrict glycolysis. PI staining demonstrated that VPS37A overexpression significantly enhanced CRC cell death under these two stress conditions in both SW620 (GA, *p* < 0.05; GD, *p* < 0.01) and DLD-1 (GA, *p* > 0.05; GD, *p* < 0.01) cells (Fig. 4H and Supplementary Fig. S2).

Furthermore, mechanistic studies revealed that VPS37A activates multiple death pathways under stress, such as enhanced autophagy flux (LC3B-II upregulation), suppressed ferroptosis resistance (Gpx4 downregulation), increased necroptotic signaling (MLKL phosphorylation), and accelerated apoptosis (PARP activation) in both SW620 and DLD-1 cells (Fig. 4I–L). These findings establish VPS37A as a context-dependent regulator of programmed cell death in CRC, particularly under metabolic stress.

### 3.5 VPS37A Suppresses NF-**κ**B Pathway Activation under Metabolic Stress

**Figure 5:**
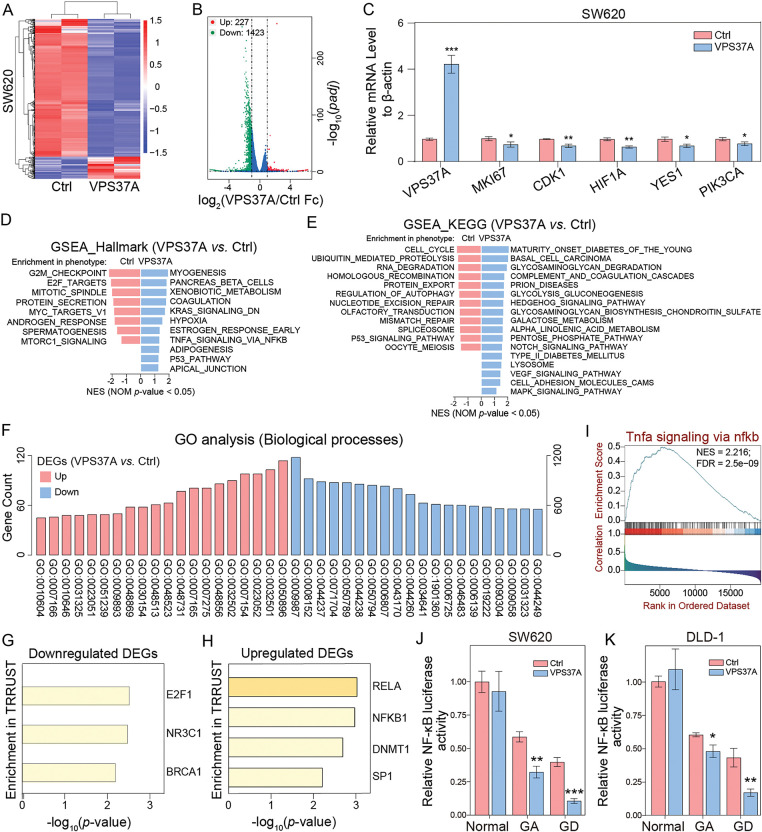
Transcriptomic and functional landscapes of VPS37A-overexpressing CRC cells. (**A**) Hierarchical clustering heatmap of differentially expressed genes (DEGs) between control and VPS37A-overexpressing SW620 cells (|log_2_FC| > 1, *p*_*adj*_ < 0.05; red: upregulated, blue: downregulated). (**B**) Volcano plot of RNA-seq data showing significant DEGs (red: upregulated, *n* = 227; green: downregulated, *n* = 1423; blue: no significant). (**C**) qRT-PCR validation of top DEGs normalized to β-actin (mean ± SD, *n* = 3). (**D**,**E**) GSEA of Hallmark and KEGG pathways. (**F**) GO analysis of biological processes enriched in DEGs. (**G**,**H**) Transcription factor (TF) enrichment analysis via TRRUST database: (**G**) TFs associated with downregulated DEGs (E2F1), (**H**) TFs linked to upregulated DEGs (NFKB1, RELA). (**I**) GSEA plot showing activation of the TNFα/NF-κB signaling pathway in VPS37A-overexpressing CRC patients. (**J**,**K**) NF-κB transcriptional activity assessed by dual-luciferase reporter assays in SW620 and DLD-1 cells under standard, galactose adaptation (GA), or glucose deprivation (GD) conditions. *p < 0.05; **p < 0.01; ***p < 0.001

To decode the tumor-suppressive regulatory network of VPS37A, we analyzed transcriptional profiles of VPS37A-overexpressing (SW620/VPS37A) and control cells (SW620/Ctrl) using RNA sequencing. Hierarchical clustering analysis of RNA-seq data revealed DEGs induced by VPS37A overexpression (Fig. 5A). DESeq2 identified 227 upregulated and 1423 downregulated genes (*p*_*adj*_ < 0.05, |log_2_FC| > 1) (Fig. 5B). qRT-PCR validation confirmed VPS37A-mediated suppression of proliferative drivers (MKI67, −26.48%, *p* < 0.05), cell cycle regulators (CDK1, −29.67%, *p* < 0.01), hypoxia-related HIF1A (−34.78%, *p* < 0.01), and PI3K/AKT signaling components (YES1, −30.24%, *p* < 0.05; PIK3CA, −20.33%, *p* < 0.05) (Fig. 5C). GSEA revealed suppression of G2/M CHECKPOINT (NES = −2.16, *p* < 0.05) and E2F TARGETS (NES = −2.02, *p* < 0.05), while KEGG analysis showed inhibition of CELL CYCLE pathways (NES = −1.77, *p* < 0.05), aligning with VPS37A’s tumor-suppressive role (Fig. 5D,E).

GO analysis linked VPS37A-regulated genes to metabolic reprogramming, with upregulated genes enriched in regulatory processes such as GO:0009893 (positive regulation of metabolic processes), GO:0031325 (positive regulation of cellular metabolism) and GO:0010604 (positive regulation of macromolecule metabolism), and downregulated DEGs primarily mapped to core metabolic functions, GO:0008152 (metabolic process), GO:0044237 (cellular metabolic process) and GO:0071704 (organic substance metabolic process) (Fig. 5F and Supplementary Table S4) indicating that the tumor-suppressive activity of VPS37A in CRC pathogenesis critically involves dynamic regulation of metabolic homeostasis.

In addition, Transcription factor (TF) enrichment analysis showed E2F1 regulation of downregulated genes, while NF-κB targets (RELA/NF-κB) were enriched among upregulated genes (Fig. 5G,H). Substantiating this finding, GSEA demonstrated robust NF-κB pathway activation upon VPS37A overexpression (Fig. 5I). This paradoxical activation of oncogenic signaling contrasts with VPS37A’s established tumor-suppressive role, suggesting that VPS37A may exert context-dependent tumor-suppressive functions in CRC through mechanisms extending beyond canonical cell cycle regulation. Dual-luciferase assays demonstrated metabolic stress-specific NF-κB suppression by VPS37A, with transcriptional activity reduced by 20.50%–73.63% under GA or GD in SW620 and DLD-1 cells, contrasting with no effect in normal conditions (Fig. 5J,K). This metabolic stress-specific suppression aligns with VPS37A’s proposed tumor-suppressive function, suggesting that VPS37A mediates context-dependent tumor suppression in CRC by differentially regulating cell death pathways under metabolic stress.

### 3.6 VPS37A Promotes the Lysosome-Mediated TNFR1 Degradation under Metabolic Stress

**Figure 6:**
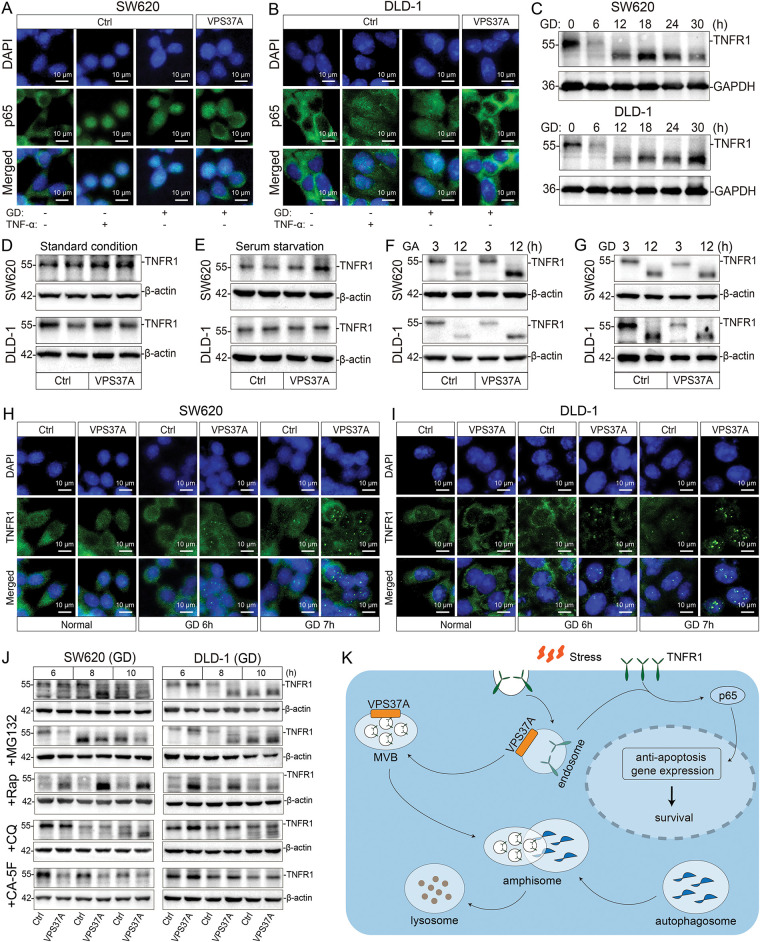
VPS37A suppresses TNFR1/NF-κB signaling under metabolic stress in CRC cells. (**A**,**B**) IF staining of p65 nuclear translocation in SW620 and DLD-1 cells under GD or TNF-α stimulation (100 ng/mL, 0.5 h). Scale bars, 10 μm. (**C**) Western blotting analysis of time-dependent TNFR1 protein degradation under GD conditions (0–30 h; GAPDH loading control). TNFR1 protein levels in VPS37A-overexpressing cells under: (**D**) standard conditions, (**E**) serum starvation, (**F**) GA (3/12 h), and (**G**) GD (3/12 h) (β-actin as loading control). (**H**,**I**) IF staining of TNFR1 subcellular localization under normal or GD conditions in SW620 and DLD-1 cells. Nuclei counterstained with DAPI. Scale bars, 10 μm. (**J**) Western blotting analysis of TNFR1 stability under GD with indicated inhibitors. β-actin as loading control. (**K**) Schematic diagram summarizing the role of VPS37A in regulating TNFR1 degradation through the endosome-lysosome-autophagy pathway, subsequently affecting p65-mediated anti-apoptotic gene expression and cellular survival under metabolic stress

To investigate how VPS37A regulates NF-κB signaling in CRC, we first analyzed p65 nuclear translocation under metabolic stress using IF. Both TNF-α stimulation and GD triggered significant p65 nuclear accumulation in SW620 and DLD-1 cells, but VPS37A overexpression strongly suppressed this process (Fig. 6A,B), indicating its role in limiting NF-κB activation. Given VPS37A’s known involvement in membrane receptor degradation via the ESCRT-1 complex, we assessed TNFR1 protein dynamics through Western blotting. Time-course experiments under GD revealed progressive TNFR1 degradation in both cell lines (Fig. 6C), suggesting metabolic stress enhances TNFR1 turnover to inactivate NF-κB signaling.

Further analysis in VPS37A-overexpressing models showed TNFR1 degradation occurred specifically under GA or GD conditions, with no effect observed under standard culture conditions or serum-starved conditions (Fig. 6D–G), establishing energy stress as a prerequisite for VPS37A-mediated regulation. IF studies revealed TNFR1 redistributed from diffuse cytoplasmic localization to punctate aggregates during GD, a pattern intensified by VPS37A overexpression (Fig. 6H,I), consistent with enhanced endocytosis and lysosomal targeting.

To dissect degradation mechanisms, pharmacological inhibition experiments demonstrated that lysosomal inhibitors (Chloroquine, CA-5F) blocked TNFR1 proteolysis, while proteasome inhibitors (MG132) showed no effect (Fig. 6J). Notably, CA-5F (a late-stage autophagy blocker) completely abolished VPS37A-dependent TNFR1 degradation (Fig. 6J), confirming autophagy-lysosomal pathways as central to this process.

These findings collectively demonstrate that VPS37A acts as a metabolic stress sensor in CRC, promoting lysosomal degradation of TNFR1 under GD to suppress NF-κB signaling. Loss of VPS37A disrupts this regulatory axis, leading to sustained NF-κB activation, impaired stress-induced cell death, and increased tumorigenic potential (Fig. 6K). This mechanism highlights the interplay between metabolic adaptation and oncogenic signaling, offering insights into therapeutic strategies targeting stress-responsive pathways in CRC.

## 4 Discussion

VPS37A is frequently lost or downregulated across a wide variety of malignancies [32], which is associated with cancer progression and poor prognosis [33–35]. Here, we conducted an integrative analysis of multi-omics data and confirmed that VPS37A is ubiquitously expressed across diverse normal tissues and is significantly downregulated in multiple malignancies, including CRC. Furthermore, both the integrative multi-omics analysis and immunohistochemical validation using our CRC patient cohort (Cohort 2) consistently confirmed that low VPS37A expression is significantly associated with advanced tumor stages and reduced overall survival in CRC. Notably, VPS37A downregulation serves as an independent prognostic biomarker predicting significantly shorter OS in CRC patients. These findings establish VPS37A as a critical regulator of cellular homeostasis and a tumor suppressor in CRC progression.

Emerging evidence highlights that the ESCRT machinery plays context-dependent roles across cancer hallmarks through its membrane-remodeling capabilities [36,37], including oncogenic signaling amplification [38], metastatic niche formation [39,40], and immune evasion [41,42], anti-cancer drug efflux [43], ultimately driving therapy resistance. The ESCRT-I complex, composed of Tumor Susceptibility Gene 101 (Tsg101), VPS37, VPS28 and Multivesicular body subunit 12 (Mvb12), serves as a pleiotropic regulator in cancer pathogenesis [44–46]. Here, we report that VPS37A, a core subunit of the ESCRT-I complex, functions as a tumor suppressor in CRC, where its loss promotes oncogenic progression through dysregulation of cell cycle control and proliferation *in vitro* and *in vivo*. Notably, while VPS37A overexpression does not significantly induce apoptosis under normal culture conditions, it surprisingly triggers programmed cell death under metabolic stress. These findings reveal that VPS37A acts as a context-dependent regulator for death signaling, suggesting druggable targets to overcome resistance to cell death during anti-tumor therapy.

NF-κB orchestrates a survival network by integrating stress adaptation, death evasion, and immune suppression across a variety of malignancies through multifaceted mechanisms. Consequently, pharmacological targeting of dysregulated NF-κB activation represents a promising therapeutic strategy to overcome therapy resistance and immune evasion [47–49]. In this study, we demonstrate that VPS37A serves as a critical context-dependent regulator of programmed cell death pathways in CRC under metabolic stress. Importantly, during metabolic stress (galactose adaptation or glucose deprivation), VPS37A not only suppresses NF-κB-driven transcriptional programs but also activates cell death, positioning VPS37A as a pleiotropic metabolic sensor that directs cancer cell fate through integration of stress-adaptive signaling.

Accumulating studies demonstrate that TNFR1, a pivotal orchestrator of canonical NF-κB signaling, functions as a molecular rheostat in cancer pathogenesis [50,51]. Dysregulation of TNFR1 degradation drives tumor immune evasion and chemoresistance [52,53]. Consequently, pharmacological inhibition of TNFR1 signaling or stabilization of TNFR1 represents a viable strategy to counteract NF-κB-driven cell death resistance [54–56]. In this study, we establish that VPS37A acts as a metabolic stress sensor in CRC and mediates TNFR1 lysosomal degradation under GD, thereby suppressing TNFR1/NF-κB signaling. VPS37A deficiency disrupts this regulatory axis, leading to sustained NF-κB activation, compromised cell death induction, and enhanced tumorigenicity. These results not only elucidate the crosstalk between metabolic stress and oncogenic signaling but also highlight potential therapeutic targets for CRC treatment. Therefore, in CRC patients with VPS37A deficiency, a multimodal therapeutic strategy could be designed to synergistically dismantle treatment resistance by orchestrating metabolic stress induction, tumor suppressor reconstitution, and oncogenic receptor degradation. Firstly, targeting glucose transporters (e.g., GLUT1) via degraders [57–59], such as GLUT1-proteolysis targeting chimeras (PROTAC) to disrupt glycolytic metabolism, thereby imposing nutrient deprivation, then restoring VPS37A functionality through tumor-localized delivery systems [60–62]—such as oncolytic adenoviruses carrying hypoxia-inducible VPS37A expression cassettes, CRISPR/Cas9 ribonucleoproteins with tumor-penetrating peptide carriers for precise gene editing, or lipid nanoparticles coated with EGFR-targeting scFv antibodies encapsulating optimized VPS37A mRNA. Alternatively, eliminating TNFR1-mediated survival signals using bifunctional degraders [63–65]—either PROTACs recruiting VHL E3 ligase for ubiquitin-proteasome disposal or lysosome-targeting chimeras (LYTACs) co-opting IGF2R for lysosomal trafficking—to terminate constitutive NF-κB activation. This integrated approach would suppress the constitutively activated NF-κB signaling—a key driver of CRC treatment resistance—by eliminating TNFR1-mediated survival signals while reinstating VPS37A-dependent tumor suppression mechanisms, ultimately synergizing to induce programmed cell death and circumvent therapeutic evasion in VPS37A-deficient CRC.

Furthermore, the observed cell death heterogeneity—encompassing apoptosis, necroptosis, and ferroptosis—suggests that VPS37A regulates a master switch integrating multiple death modalities, potentially through crosstalk with mitochondrial integrity or redox homeostasis. Additionally, the contribution of alternative pathways such as ubiquitination or chaperone-mediated autophagy warrants investigation. The *in vivo* relevance of the VPS37A-NF-κB axis in metastatic niches and therapy response requires validation using genetically engineered models and patient-derived xenografts. Critically, future studies exploring small-molecule enhancers of VPS37A activity or lysosomal TNFR1 degradation could accelerate the translation of these mechanistic insights into clinical applications.

This study also has several limitations. First, although VPS37A interacts with other subunits of the ESCRT-I complex, it remains unclear whether the observed suppression of malignant phenotypes caused by VPS37A overexpression is mediated through functional modulation of the ESCRT-I complex in CRC. Second, both RNA-seq analysis of VPS37A-overexpressing cells and TCGA bioinformatics data reveal a significant positive correlation between VPS37A expression and NF-κB signaling activation. However, this finding conflicts with the known tumor-suppressive role of VPS37A in CRC, and the underlying mechanism of this paradox remains unclear. Furthermore, the specificity of glucose deprivation (vs. general nutrient stress) in triggering VPS37A-dependent TNFR1 degradation warrants deeper exploration, including testing TNFα-stimulated degradation. Finally, while multiple death pathways were implicated, genetic/pharmacological dissection of their relative contributions was lacking. These limitations underscore the necessity of employing orthogonal approaches, such as knockout models, colocalization studies with endosomal-lysosomal markers, and *in vivo* metabolic stress paradigms, to validate the proposed mechanism and its therapeutic relevance.

## 5 Conclusion

This study delineates a novel VPS37A-TNFR1-NF-κB regulatory axis that governs metabolic stress adaptation in CRC. Our discovery of VPS37A-mediated TNFR1 degradation under metabolic stress provides mechanistic insight into NF-κB regulation in nutrient-deprived tumors. Restoring VPS37A function through oncolytic adenoviruses, CRISPR/Cas9 ribonucleoproteins for precise gene editing, or lipid nanoparticles encapsulating optimized VPS37A mRNA or mimicking its degradative activity on TNFR1 using PROTACs for ubiquitin-proteasome disposal or LYTACs for lysosomal trafficking may represent promising therapeutic avenues for CRC, particularly in advanced-stage tumors where metabolic stress and NF-κB hyperactivation converge to drive lethality.

## Supplementary Materials



## Data Availability

The data that support the findings of this study are available from the corresponding author upon reasonable request.

## Supplementary Materials

The supplementary material is available online at https://www.techscience.com/doi/10.32604/or.2025.065739/s1.

## References

[1] Siegel RL, Giaquinto AN, Jemal A. Cancer statistics. 2024 CA Cancer J Clin. 2024;74(1):12–49. doi:10.3322/caac.21718. 38230766

[2] Shin AE, Giancotti FG, Rustgi AK. Metastatic colorectal cancer: mechanisms and emerging therapeutics. Trends Pharmacol Sci. 2023;44(4):222–36. doi:10.1016/j.tips.2023.01.003; 36828759 PMC10365888

[3] Bray F, Laversanne M, Sung H, Ferlay J, Siegel RL, Soerjomataram I, et al. Global cancer statistics 2022: gLOBOCAN estimates of incidence and mortality worldwide for 36 cancers in 185 countries. CA Cancer J Clin. 2024;74(3):229–63. doi:10.3322/caac.21660; 38572751

[4] Lin Z, Wan AH, Sun L, Liang H, Niu Y, Deng Y, et al. N6-methyladenosine demethylase FTO enhances chemo-resistance in colorectal cancer through SIVA1-mediated apoptosis. Mol Ther. 2023;31(2):517–34. doi:10.1016/j.ymthe.2022.10.012; 36307991 PMC9931553

[5] Di Grazia A, Marafini I, Pedini G, Di Fusco D, Laudisi F, Dinallo V, et al. The fragile X mental retardation protein regulates RIPK1 and colorectal cancer resistance to necroptosis. Cell Mol Gastroenterol Hepatol. 2021;11(2):639–58. doi:10.1016/j.jcmgh.2020.10.009; 33091622 PMC7806864

[6] Lan H, Liu Y, Liu J, Wang X, Guan Z, Du J, et al. Tumor-associated macrophages promote oxaliplatin resistance via METTL3-Mediated m6A of TRAF5 and necroptosis in colorectal cancer. Mol Pharm. 2021;18(3):1026–37. doi:10.1021/acs.molpharmaceut.0c00961; 33555197

[7] Song M, Huang S, Wu X, Zhao Z, Liu X, Wu C, et al. UBR5 mediates colorectal cancer chemoresistance by attenuating ferroptosis via Lys 11 ubiquitin-dependent stabilization of Smad3-SLC7A11 signaling. Redox Biol. 2024;76(9–14 12 Suppl 11):103349. doi:10.1016/j.redox.2024.103349; 39260061 PMC11415886

[8] Zhang Q, Deng T, Zhang H, Zuo D, Zhu Q, Bai M, et al. Adipocyte-derived exosomal MTTP suppresses ferroptosis and promotes chemoresistance in colorectal cancer. Adv Sci. 2022;9(28):e2203357. doi:10.1002/advs.202203357; 35978266 PMC9534973

[9] Wang N, Zhang L, Leng XX, Xie YL, Kang ZR, Zhao LC, et al. Fusobacterium nucleatum induces chemoresistance in colorectal cancer by inhibiting pyroptosis via the Hippo pathway. Gut Microbes. 2024;16(1):2333790. doi:10.1080/19490976.2024.2333790; 38533566 PMC10978024

[10] Manzoor S, Saber-Ayad M, Maghazachi AA, Hamid Q, Muhammad JS. MLH1 mediates cytoprotective nucleophagy to resist 5-Fluorouracil-induced cell death in colorectal carcinoma. Neoplasia. 2022;24(2):76–85. doi:10.1016/j.neo.2021.12.003; 34952246 PMC8695220

[11] Ghasemian A, Omear HA, Mansoori Y, Mansouri P, Deng X, Darbeheshti F, et al. Long non-coding RNAs and JAK/STAT signaling pathway regulation in colorectal cancer development. Front Genet. 2023;14:1297093. doi:10.3389/fgene.2023.1297093; 38094755 PMC10716712

[12] Bhattacharyya S, Mandal D, Sen GS, Pal S, Banerjee S, Lahiry L, et al. Tumor-induced oxidative stress perturbs nuclear factor-kappaB activity-augmenting tumor necrosis factor-alpha-mediated T-cell death: protection by curcumin. Cancer Res. 2007;67(1):362–70. doi:10.1158/0008-5472.can-06-2583; 17210719

[13] Chen J, Stark LA. Insights into the Relationship between Nucleolar Stress and the NF-kappaB Pathway. Trends Genet. 2019;35(10):768–80. doi:10.1016/j.tig.2019.07.009; 31434627

[14] Capece D, D’Andrea D, Begalli F, Goracci L, Tornatore L, Alexander JL, et al. Enhanced triacylglycerol catabolism by carboxylesterase 1 promotes aggressive colorectal carcinoma. J Clin Investig. 2021;131(11):e137845. doi:10.1172/jci137845; 33878036 PMC8159693

[15] Mauro C, Leow SC, Anso E, Rocha S, Thotakura AK, Tornatore L, et al. NF-kappaB controls energy homeostasis and metabolic adaptation by upregulating mitochondrial respiration. Nat Cell Biol. 2011;13(10):1272–9. doi:10.1038/ncb2324; 21968997 PMC3462316

[16] Wang X, Liu R, Qu X, Yu H, Chu H, Zhang Y, et al. Alpha-Ketoglutarate-Activated NF-kappaB signaling promotes compensatory glucose uptake and brain tumor development. Mol Cell. 2019;76(1):148–62. doi:10.1016/j.molcel.2019.07.007; 31447391

[17] Tan G, Wu L, Tan J, Zhang B, Tai WC, Xiong S, et al. MiR-1180 promotes apoptotic resistance to human hepatocellular carcinoma via activation of NF-kappaB signaling pathway. Sci Rep. 2016;6(1):22328. doi:10.1038/srep22328; 26928365 PMC4772113

[18] Karin M, Greten FR. NF-kappaB: linking inflammation and immunity to cancer development and progression. Nat Rev Immunol. 2005;5(10):749–59. doi:10.1038/nri1703; 16175180

[19] Pikarsky E, Porat RM, Stein I, Abramovitch R, Amit S, Kasem S, et al. NF-kappaB functions as a tumour promoter in inflammation-associated cancer. Nature. 2004;431(7007):461–6. doi:10.1038/nature02924; 15329734

[20] Xu R, Du A, Deng X, Du W, Zhang K, Li J, et al. tsRNA-GlyGCC promotes colorectal cancer progression and 5-FU resistance by regulating SPIB. J Exp Clin Cancer Res. 2024;43(1):230. doi:10.1186/s13046-024-03132-6; 39153969 PMC11330149

[21] Dondelinger Y, Jouan-Lanhouet S, Divert T, Theatre E, Bertin J, Gough PJ, et al. NF-kappaB-independent role of IKKalpha/IKKbeta in preventing RIPK1 kinase-dependent apoptotic and necroptotic cell death during TNF signaling. Mol Cell. 2015;60(1):63–76. doi:10.1016/j.molcel.2015.07.032; 26344099

[22] Micheau O, Tschopp J. Induction of TNF receptor I-mediated apoptosis via two sequential signaling complexes. Cell. 2003;114(2):181–90. doi:10.1016/s0092-8674(03)00521-x; 12887920

[23] Li Y, Ye R, Dai H, Lin J, Cheng Y, Zhou Y, et al. Exploring TNFR1: from discovery to targeted therapy development. J Transl Med. 2025;23(1):71. doi:10.1186/s12967-025-06122-0; 39815286 PMC11734553

[24] Yang Y, Sun M, Yao W, Wang F, Li X, Wang W, et al. Compound kushen injection relieves tumor-associated macrophage-mediated immunosuppression through TNFR1 and sensitizes hepatocellular carcinoma to sorafenib. J Immunother Cancer. 2020;8(1):e000317. doi:10.1136/jitc-2019-000317corr1; 32179631 PMC7073790

[25] Takahashi Y, Liang X, Hattori T, Tang Z, He H, Chen H, et al. VPS37A directs ESCRT recruitment for phagophore closure. J Cell Biol. 2019;218(10):3336–54. doi:10.1083/jcb.201902170; 31519728 PMC6781443

[26] Stefani F, Zhang L, Taylor S, Donovan J, Rollinson S, Doyotte A, et al. UBAP1 is a component of an endosome-specific ESCRT-I complex that is essential for MVB sorting. Curr Biol. 2011;21(14):1245–50. doi:10.1016/j.cub.2011.06.028; 21757351

[27] Ye Y, Liang X, Wang G, Bewley MC, Hamamoto K, Liu X, et al. Identification of membrane curvature sensing motifs essential for VPS37A phagophore recruitment and autophagosome closure. Commun Biol. 2024;7(1):334. doi:10.1038/s42003-024-06026-7; 38491121 PMC10942982

[28] Tomasich E, Topakian T, Heller G, Udovica S, Krainer M, Marhold M. Loss of HCRP1 leads to upregulation of PD-L1 via STAT3 activation and is of prognostic significance in EGFR-dependent cancer. Transl Res. 2021;230:21–33. doi:10.1016/j.trsl.2020.11.005; 33197651

[29] Lu Y, Li Z, Zhang S, Zhang T, Liu Y, Zhang L. Cellular mitophagy: mechanism, roles in diseases and small molecule pharmacological regulation. Theranostics. 2023;13(2):736–66. doi:10.7150/thno.79876; 36632220 PMC9830443

[30] Liu Y, Zhao R, Wei Y, Li M, Wang H, Niu W, et al. BRD7 expression and c-Myc activation forms a double-negative feedback loop that controls the cell proliferation and tumor growth of nasopharyngeal carcinoma by targeting oncogenic miR-141. J Exp Clin Cancer Res. 2018;37(1):64. doi:10.1186/s13046-018-0734-2; 29559001 PMC5859396

[31] Zhao R, Liu Y, Wang H, Yang J, Niu W, Fan S, et al. BRD7 plays an anti-inflammatory role during early acute inflammation by inhibiting activation of the NF-κB signaling pathway. Cell Mol Immunol. 2017;14(10):830–41. doi:10.1038/cmi.2016.31; 27374794 PMC5649105

[32] Kolmus K, Erdenebat P, Szymańska E, Stewig B, Goryca K, Derezińska-Wołek E, et al. Concurrent depletion of Vps37 proteins evokes ESCRT-I destabilization and profound cellular stress responses. J Cell Sci. 2021;134(1):jcs250951. doi:10.1101/2020.07.02.183954. 33419951

[33] Chen F, Zhang L, Wu J, Huo F, Ren X, Zheng J, et al. HCRP-1 regulates EGFR-AKT-BIM-mediated anoikis resistance and serves as a prognostic marker in human colon cancer. Cell Death Dis. 2018;9(12):1176. doi:10.1038/s41419-018-1217-2; 30518879 PMC6281589

[34] Sun L, Lü J, Ding S, Bi D, Ding K, Niu Z, et al. HCRP1 regulates proliferation, invasion, and drug resistance via EGFR signaling in prostate cancer. Biomed Pharmacother. 2017;91:202–7. doi:10.1016/j.biopha.2017.04.040; 28458158

[35] Wu Y, Yang Y, Xian YS. HCRP1 inhibits cell proliferation and invasion and promotes chemosensitivity in esophageal squamous cell carcinoma. Chem Biol Interact. 2019;308:357–63. doi:10.1016/j.cbi.2019.05.032; 31152734

[36] Hurley JH. ESCRT complexes and the biogenesis of multivesicular bodies. Curr Opin Cell Biol. 2008;20(1):4–11. doi:10.1016/j.ceb.2007.12.002; 18222686 PMC2282067

[37] Vietri M, Radulovic M, Stenmark H. The many functions of ESCRTs. Nat Rev Mol Cell Biol. 2020;21(1):25–42. doi:10.1038/s41580-019-0177-4; 31705132

[38] Al-Nedawi K, Meehan B, Micallef J, Lhotak V, May L, Guha A, et al. Intercellular transfer of the oncogenic receptor EGFRvIII by microvesicles derived from tumour cells. Nat Cell Biol. 2008;10(5):619–24. doi:10.1038/ncb1725; 18425114

[39] Hoshino A, Costa-Silva B, Shen TL, Rodrigues G, Hashimoto A, Tesic Mark M, et al. Tumour exosome integrins determine organotropic metastasis. Nature. 2015;527(7578):329–35. doi:10.1038/nature15756; 26524530 PMC4788391

[40] Peinado H, Zhang H, Matei IR, Costa-Silva B, Hoshino A, Rodrigues G, et al. Pre-metastatic niches: organ-specific homes for metastases. Nat Rev Cancer. 2017;17(5):302–17. doi:10.1038/nrc.2017.6; 28303905

[41] Daassi D, Mahoney KM, Freeman GJ. The importance of exosomal PDL1 in tumour immune evasion. Nat Rev Immunol. 2020;20(4):209–15. doi:10.1038/s41577-019-0264-y; 31965064

[42] Chen Y, Liang J, Chen S, Lin N, Xu S, Miao J, et al. Discovery of vitexin as a novel VDR agonist that mitigates the transition from chronic intestinal inflammation to colorectal cancer. Mol Cancer. 2024;23(1):196. doi:10.1186/s12943-024-02108-6; 39272040 PMC11395219

[43] Safaei R, Larson BJ, Cheng TC, Gibson MA, Otani S, Naerdemann W, et al. Abnormal lysosomal trafficking and enhanced exosomal export of cisplatin in drug-resistant human ovarian carcinoma cells. Mol Cancer Ther. 2005;4(10):1595–604. doi:10.1158/1535-7163.mct-05-0102; 16227410

[44] Chu T, Sun J, Saksena S, Emr SD. New component of ESCRT-I regulates endosomal sorting complex assembly. J Cell Biol. 2006;175(5):815–23. doi:10.1083/jcb.200608053; 17145965 PMC2064680

[45] Henne WM, Buchkovich NJ, Emr SD. The ESCRT pathway. Dev Cell. 2011;21(1):77–91. doi:10.1016/j.devcel.2011.05.015; 21763610

[46] Katzmann DJ, Babst M, Emr SD. Ubiquitin-dependent sorting into the multivesicular body pathway requires the function of a conserved endosomal protein sorting complex, ESCRT-I. Cell. 2001;106(2):145–55. doi:10.1016/s0092-8674(01)00434-2; 11511343

[47] Guo Q, Jin Y, Chen X, Ye X, Shen X, Lin M, et al. NF-kappaB in biology and targeted therapy: new insights and translational implications. Signal Transduct Target Ther. 2024;9(1):53. doi:10.1038/s41392-024-01757-9; 38433280 PMC10910037

[48] Yu H, Lin L, Zhang Z, Zhang H, Hu H. Targeting NF-kappaB pathway for the therapy of diseases: mechanism and clinical study. Signal Transduct Target Ther. 2020;5(1):209. doi:10.1038/s41392-020-00312-6; 32958760 PMC7506548

[49] Liang J, Dai W, Liu C, Wen Y, Chen C, Xu Y, et al. Gingerenone A Attenuates ulcerative colitis via targeting IL-17RA to Inhibit inflammation and restore intestinal barrier function. Adv Sci. 2024;11(28):e2400206. doi:10.1002/advs.202400206; 38639442 PMC11267284

[50] Chan SH, Kuo WH, Wang LH. SCEL regulates switches between pro-survival and apoptosis of the TNF-alpha/TNFR1/NF-kappaB/c-FLIP axis to control lung colonization of triple negative breast cancer. J Biomed Sci. 2023;30(1):93. doi:10.1186/s12929-023-00986-4; 38037106 PMC10688137

[51] Shi G, Hu Y. TNFR1 and TNFR2, which link NF-kappaB activation, drive lung cancer progression, cell dedifferentiation, and metastasis. Cancers. 2023;15(17):4299. doi:10.3390/cancers15174299; 37686574 PMC10487001

[52] Bai J, Sui J, Demirjian A, Vollmer CM Jr., Marasco W, Callery MP. Predominant Bcl-XL knockdown disables antiapoptotic mechanisms: tumor necrosis factor-related apoptosis-inducing ligand-based triple chemotherapy overcomes chemoresistance in pancreatic cancer cells *in vitro*. Cancer Res. 2005;65(6):2344–52. doi:10.1158/0008-5472.can-04-3502; 15781649

[53] Tang L, Zhang H, Zhou F, Wei Q, Du M, Wu J, et al. Targeting autophagy overcomes cancer-intrinsic resistance to CAR-T immunotherapy in B-cell malignancies. Cancer Commun. 2024;44(3):408–32. doi:10.1002/cac2.12525; 38407943 PMC10958674

[54] Meskyte EM, Pezze L, Bartolomei L, Forcato M, Bocci IA, Bertalot G, et al. ETV7 reduces inflammatory responses in breast cancer cells by repressing the TNFR1/NF-kappaB axis. Cell Death Dis. 2023;14(4):263. doi:10.21203/rs.3.rs-2067615/v1. 37041130 PMC10089821

[55] Xu H, Liu T, Li J, Chen F, Xu J, Hu L, et al. Roburic acid targets TNF to inhibit the NF-κB signaling pathway and suppress human colorectal cancer cell growth. Front Immunol. 2022;13:853165. doi:10.3389/fimmu.2022.853165; 35222445 PMC8864141

[56] Xia Y, Huang P, Qian YY, Wang Z, Jin N, Li X, et al. PARP inhibitors enhance antitumor immune responses by triggering pyroptosis via TNF-caspase 8-GSDMD/E axis in ovarian cancer. J Immunother Cancer. 2024;12(10):e009032. doi:10.1136/jitc-2024-009032; 39366751 PMC11459312

[57] Li Y, Tang S, Shi X, Lv J, Wu X, Zhang Y, et al. Metabolic classification suggests the GLUT1/ALDOB/G6PD axis as a therapeutic target in chemotherapy-resistant pancreatic cancer. Cell Rep Med. 2023;4(9):101162. doi:10.1016/j.xcrm.2023.101162; 37597521 PMC10518604

[58] Wang Y, Wang X, Bai B, Shaha A, He X, He Y, et al. Targeting Src SH3 domain-mediated glycolysis of HSC suppresses transcriptome, myofibroblastic activation, and colorectal liver metastasis. Hepatology. 2024;80(3):578–94. doi:10.1097/hep.0000000000000763; 38271673 PMC11266532

[59] Wang K, Dai X, Yu A, Feng C, Liu K, Huang L. Peptide-based PROTAC degrader of FOXM1 suppresses cancer and decreases GLUT1 and PD-L1 expression. J Exp Clin Cancer Res. 2022;41(1):289. doi:10.1186/s13046-022-02483-2; 36171633 PMC9520815

[60] Jia Y, Wang X, Li L, Li F, Zhang J, Liang XJ. Lipid nanoparticles optimized for targeting and release of nucleic acid. Adv Mater. 2024;36(4):e2305300. doi:10.1002/adma.202305300; 37547955

[61] Wei T, Cheng Q, Min YL, Olson EN, Siegwart DJ. Systemic nanoparticle delivery of CRISPR-Cas9 ribonucleoproteins for effective tissue specific genome editing. Nat Commun. 2020;11(1):3232. doi:10.1038/s41467-020-17029-3; 32591530 PMC7320157

[62] Xie D, Tian Y, Hu D, Wang Y, Yang Y, Zhou B, et al. Oncolytic adenoviruses expressing checkpoint inhibitors for cancer therapy. Signal Transduct Target Ther. 2023;8(1):436. doi:10.1038/s41392-023-01683-2; 38016957 PMC10684539

[63] Diehl CJ, Ciulli A. Discovery of small molecule ligands for the von Hippel-Lindau (VHL) E3 ligase and their use as inhibitors and PROTAC degraders. Chem Soc Rev. 2022;51(19):8216–57. doi:10.1016/j.ejmech.2023.116041; 35983982 PMC9528729

[64] Pan Y, Xiang Q, Deng K, Anwar MI, Wang L, Wang Y, et al. Engineering IGF2 for Lysosome-targeting chimeras development to target drug-resistant membrane proteins in tumor therapy. Protein Sci. 2025;34(3):e70051. doi:10.1002/pro.70051; 39969096 PMC11837023

[65] Yu S, Shi T, Li C, Xie C, Wang F, Liu X. Programming DNA nanoassemblies into polyvalent lysosomal degraders for potent degradation of pathogenic membrane proteins. Nano Lett. 2024;24(37):11573–80. doi:10.1021/acs.nanolett.4c03102; 39225423

