# Supplementary material for: VPS37A Activates the Autophagy-Lysosomal Pathway for TNFR1 Degradation and Induces NF-κB-Regulated Cell Death under Metabolic Stress in Colorectal Cancer
Source: Oncol Res. 2025 Jul 18;33(8):2085–105. doi: 10.32604/or.2025.065739 (PMC12308250; doi:10.32604/or.2025.065739)
Supplement: Supplementary file 1 [file OncolRes-33-65739-s001.docx]

**Supplementary Figure S1**


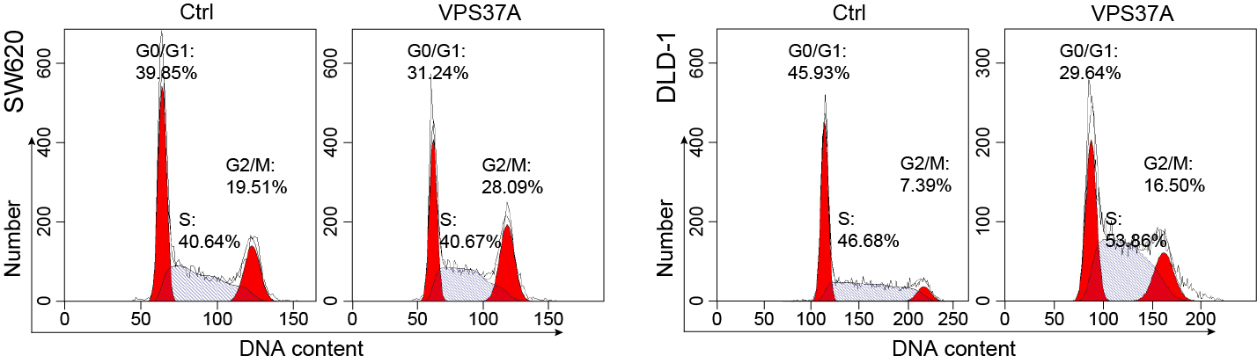


**Supplementary Figure S1.** VPS37A overexpression disrupts cell cycle progression in CRC cells. Flow cytometric quantification of cell cycle phases (G0/G1, S, G2/M) in control and VPS37A-overexpressing cells using PI-based DNA staining.

**Supplementary Figure S2**


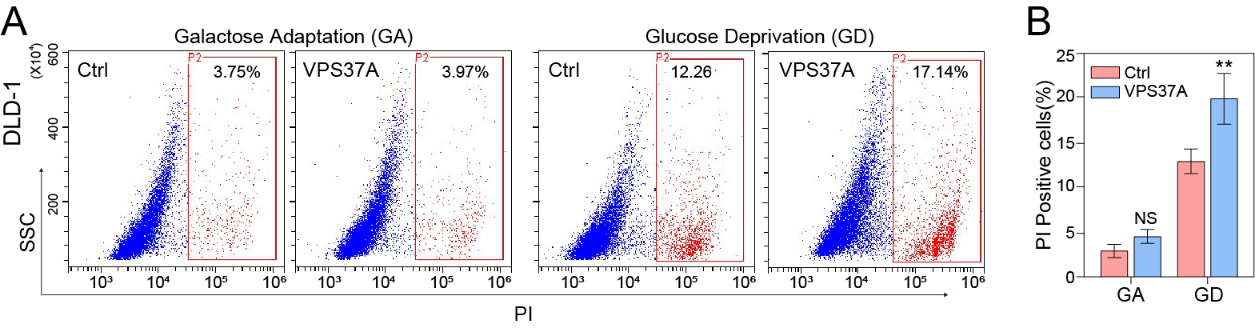


**Supplementary Figure S2**. VPS37A overexpression induces cell death in colorectal cancer (CRC) cells under metabolic stress conditions. (A) Flow cytometry analysis of PI-positive cells (indicative of late apoptosis/necrosis) in VPS37A-overexpressing and control cells cultured under galactose adaptation (GA) or glucose deprivation (GD). (B) Quantification of cell death (mean ± SD, n=3; NS, not significant; ***p* < 0.01)

**Supplementary Table S1. The expression levels of the VPS37A protein in both normal tissue and the corresponding paired tumor tissue specimens.**

| **Patient ID** | **Characteristics** | | | **VPS37A protein level (IHC intensity)** | |
| --- | --- | --- | --- | --- | --- |
|  | **Sex** | **Age** | **location** | **paired tumor tissue** | **Normal tissue** |
| P2 | female | 71 | colon | 2 | 1 |
| P3 | male | 31 | colon | 2 | 0 |
| P4 | female | 33 | colon | 2 | 3 |
| P5 | female | 40 | colon | 2 | 0 |
| P6 | female | 57 | rectum | 2 | 3 |
| P9 | male | 56 | rectum | 1 | 2 |
| P11 | male | 56 | colon | 2 | 3 |
| P13 | female | 46 | colon | 1 | 2 |
| P14 | male | 61 | rectum | 0 | 1 |
| P15 | male | 74 | colon | 0 | 0 |
| P18 | female | 41 | rectum | 1 | 2 |
| P19 | male | 68 | colon | 2 | 1 |
| P21 | male | 51 | rectum | 1 | 3 |
| P22 | male | 55 | colon | 3 | 3 |
| P25 | female | 64 | rectum | 2 | 1 |
| P28 | female | 58 | colon | 3 | 3 |
| P30 | male | 50 | rectum | 1 | 3 |
| P37 | male | 73 | colon | 0 | 1 |
| P44 | male | 73 | colon | 0 | 2 |
| P46 | male | 54 | rectum | 1 | 3 |
| P47 | female | 63 | colon | 1 | 2 |
| P51 | female | 39 | rectum | 3 | 3 |
| P60 | female | 55 | colon | 3 | 3 |
| P64 | female | 60 | rectum | 0 | 1 |
| P66 | male | 63 | rectum | 1 | 2 |
| P75 | female | 56 | colon | 2 | 3 |
| P83 | male | 49 | rectum | 1 | 2 |
| P102 | male | 52 | rectum | 1 | 2 |
| P115 | female | 63 | rectum | 1 | 2 |
| P120 | female | 47 | rectum | 1 | 2 |

**Supplementary Table S2. Primer pair sequences for qRT-PCR**

| Gene Symbol | Transcript ID | Sequence |
| --- | --- | --- |
| VPS37A | NM_152415.3 | Forward: 5’-TGCACATGAAGCTGAGGAAG-3' |
|  |  | Reverse: 5’-TTGGCTGTGCATTGCTATCG-3' |
| MKI67 | NM_002417.5 | Forward: 5’-GAAAGAGTGGCAACCTGCCTTC-3' |
|  |  | Reverse: 5’-GCACCAAGTTTTACTACATCTGCC-3' |
| CDK1 | NM_001786.5 | Forward: 5’-AGTCTACGGGCTACCCGATT-3' |
|  |  | Reverse: 5’-CCACTCTGCCCTAGGCTTTC-3' |
| HIF1A | NM_001530.4 | Forward: 5’-TATGAGCCAGAAGAACTTTTAGGC-3' |
|  |  | Reverse: 5’-CACCTCTTTTGGCAAGCATCCTG-3' |
| YES1 | NM_005433.4 | Forward: 5’-GTTTCGGCGAAGTGTGGATG-3' |
|  |  | Reverse: 5’-TCACCATACCTTGTCTTGCTGT-3' |
| PIK3CA | NM_006218.4 | Forward: 5’-GGACCCGATGCGGTTAGAG-3' |
|  |  | Reverse: 5’-ATCAAGTGGATGCCCCACAG-3' |
| ACTB | NM_001101.5 | Forward: 5’-TCACCAACTGGGACGACATG-3' |
|  |  | Reverse: 5’-GTCACCGGAGTCCATCACGAT-3' |

**Supplementary Table S3. Antibodies for Western Blotting Analysis**

| Antibodies Name | Brand | Dilution | Lot# |
| --- | --- | --- | --- |
| VPS37A | Santa Cruz | 1:500 | sc-376978 |
| Flag | Sigma-Aldrich | 1:2000 | F1804 |
| LC3B | Proteintech | 1:1000 | 14600-1-AP |
| P62 | Proteintech | 1:1000 | 18420-1-AP |
| Gpx4 | Santa Cruz | 1:1000 | sc-166437 |
| MLKL | Santa Cruz | 1:1000 | sc-293201 |
| p-MLKL | Affinity | 1:1000 | AF7420 |
| PARP | Proteintech | 1:1000 | 13371-1-AP |
| cPARP, cleaved PARP (Asp214) | Cell Signal Technology | 1:1000 | #9541 |
| TNFR1 | Santa Cruz | 1:400 | sc-8436 |
| GAPDH | Proteintech | 1:5000 | 60004-1-Ig |
| β-actin | Proteintech | 1:5000 | 66009-1-Ig |
| HRP-conjugated Goat Anti-Rabbit IgG(H+L) | Proteintech | 1:5000 | SA00001-2 |
| HRP-conjugated Goat Anti-Mouse IgG(H+L) | Proteintech | 1:5000 | SA00001-1 |

**Supplementary Table S4. GO analysis (Biological processes) of DEGs**

| Accession | Name | Down DEGs Count |
| --- | --- | --- |
| GO:0009987 | cellular process | 1179 |
| GO:0008152 | metabolic process | 922 |
| GO:0044237 | cellular metabolic process | 885 |
| GO:0071704 | organic substance metabolic process | 876 |
| GO:0050789 | regulation of biological process | 875 |
| GO:0044238 | primary metabolic process | 856 |
| GO:0050794 | regulation of cellular process | 844 |
| GO:0006807 | nitrogen compound metabolic process | 834 |
| GO:0043170 | macromolecule metabolic process | 802 |
| GO:0044260 | cellular macromolecule metabolic process | 733 |
| GO:0034641 | cellular nitrogen compound metabolic process | 629 |
| GO:1901360 | organic cyclic compound metabolic process | 614 |
| GO:0006725 | cellular aromatic compound metabolic process | 603 |
| GO:0046483 | heterocycle metabolic process | 602 |
| GO:0006139 | nucleobase-containing compound metabolic process | 592 |
| GO:0019222 | regulation of metabolic process | 580 |
| GO:0090304 | nucleic acid metabolic process | 561 |
| GO:0031323 | regulation of cellular metabolic process | 558 |
| GO:0009058 | biosynthetic process | 558 |
| GO:0044249 | cellular biosynthetic process | 553 |
| Accession | Name | Up DEGs Count |
| GO:0050896 | response to stimulus | 114 |
| GO:0032501 | multicellular organismal process | 103 |
| GO:0023052 | signaling | 98 |
| GO:0007154 | cell communication | 98 |
| GO:0032502 | developmental process | 90 |
| GO:0048856 | anatomical structure development | 86 |
| GO:0007275 | multicellular organism development | 81 |
| GO:0007165 | signal transduction | 81 |
| GO:0048731 | system development | 77 |
| GO:0048523 | negative regulation of cellular process | 63 |
| GO:0048513 | animal organ development | 61 |
| GO:0030154 | cell differentiation | 58 |
| GO:0048869 | cellular developmental process | 58 |
| GO:0009893 | positive regulation of metabolic process | 50 |
| GO:0051239 | regulation of multicellular organismal process | 49 |
| GO:0023051 | regulation of signaling | 49 |
| GO:0031325 | positive regulation of cellular metabolic process | 48 |
| GO:0010646 | regulation of cell communication | 48 |
| GO:0007166 | cell surface receptor signaling pathway | 46 |
| GO:0010604 | positive regulation of macromolecule metabolic process | 45 |

**Supplementary Table S5. Differentially Expressed Genes (DEGs) Identified by Gene Set Enrichment Analysis (GSEA)**

| **Pathway** | **Set Size** | **DEGs ID** |
| --- | --- | --- |
| Cell cycle | 124 | 10393/1022/1647/5000/4193/7533/1026/8697/4087/9184/4089/7042/990/7029/9978/4085/8317/1017/699/890/891/494551/996/85417/2932/5591/902/983/8555/6502/4999/10274/29945 |
| Apoptosis | 87 | 5533/8793/8795/8797/330/1147/3556/1439/836/5534/355/840/5573/843/3552/5294/51135/317/5532/5577/841/8794/329/3553/356/3554/1676/8503/5530/581/5293/11213/7132/8743/5290/5613/4790/8837 |
| G2m checkpoint | 199 | 55872/7486/10772/5926/9994/4331/8819/3091/1810/9184/1062/51155/990/22823/10762/57082/10733/3015/4085/6632/10403/8317/51203/699/22809/10592/10460/890/3161/996/8065/10721/3364/904/26271/3312/9252/983/7468/4212/3832/10274/6118/6491/262/9493/7112/6760/5558/10664/7514/9156/10926/1164/9055/4751/81539/8899/4853/8318/9212/51659/3619/1033/3837/3838/332/5371/1019/1950/27338/6558/4502 |
| Apoptosis | 161 | 665/2936/5366/330/10950/1960/837/1806/3606/836/1647/5534/3459/969/2232/355/301/840/2643/1026/834/3552/6648/7042/2107/7417/6093/841/1017/8915/2026/10059/3553/356/6309/1491/3082/1676/3669/3569/960/3162/6303/552/5551/9890/3659/6574/581/467/4739/3301/914/6041/650/8743/2012/929/2149/8837/4170 |
| Oxidative phosphorylation | 200 | 4724/1355/36/291/4729/4190/840/4976/3032/1737/211/34/2110/1353/1352/81689/8050/4329/5018/10651/56945/6390/4700/10935/1738/51318/518/7417/2108/80273/1350/56993/51382/4701/1347/10476/23479/64960/9550/10884/5435/64963/4712/4942/10449/10440/10975/22/8604/23203/4717/3052/581/1743/4259/6832/529/2271/7386/4967/7388/4702/515/522/506/2746/4129/528/3419/4720/10312/8803/2395/4719/23530/8992/3945/64981/4698/3418/29796/2230/26520/4711/55288/4835/38/10128/4695 |
| Glycolysis | 200 | 5516/55276/10776/3796/178/4190/55635/5917/5223/8819/112399/2530/9653/5033/10370/2585/1738/54541/10380/4200/2745/7039/7852/2026/51129/6781/1491/79644/3161/3669/9435/11015/136/58528/2729/960/7360/9123/23443/5318/11319/3309/8460/9276/983/54982/283/4351/3099/8974/26035/2719/4907/9456/5214/6383/23036 |
| Tnfa signaling via nfkb | 200 | 25816/1846/8744/11080/330/10950/10769/1960/490/24145/55332/3606/1647/5791/969/1326/4783/4084/4929/2643/6373/1026/10010/5055/23764/5142/25976/5209/1880/7097/3552/5743/3572/64135/6648/4780/51278/2150/1847/7050/694/3433/127544/5806/7130/3575/3976/5329/22822/3949/604/2152/329/3553/182/3627/5966/6372/2921/9120/5271/10135/6351/2919/3569/687/19/2920/960/6303/8303/3601/3659/2353/9021/6446/1827/4791/467/10725/9945/9314/2355/3164/3624/23645/650/5328/1959/6364/8767/3398/8553/4790/23258/8837/4170/5734/79693/8942/150094/3604/3383/388/10365/23308/597/6352/3371 |

**Supplementary Table S6. Annotation of Cancer Types in The Cancer Genome Atlas (TCGA)**

| **TCGA** | **Detail** |
| --- | --- |
| ACC | Adrenocortical carcinoma |
| BLCA | Bladder Urothelial Carcinoma |
| BRCA | Breast invasive carcinoma |
| CESC | Cervical squamous cell carcinoma and endocervical adenocarcinoma |
| CHOL | Cholangio carcinoma |
| COAD | Colon adenocarcinoma |
| DLBC | Lymphoid Neoplasm Diffuse Large B-cell Lymphoma |
| ESCA | Esophageal carcinoma |
| GBM | Glioblastoma multiforme |
| HNSC | Head and Neck squamous cell carcinoma |
| KICH | Kidney Chromophobe |
| KIRC | Kidney renal clear cell carcinoma |
| KIRP | Kidney renal papillary cell carcinoma |
| LAML | Acute Myeloid Leukemia |
| LGG | Brain Lower Grade Glioma |
| LIHC | Liver hepatocellular carcinoma |
| LUAD | Lung adenocarcinoma |
| LUSC | Lung squamous cell carcinoma |
| MESO | Mesothelioma |
| OV | Ovarian serous cystadenocarcinoma |
| PAAD | Pancreatic adenocarcinoma |
| PCPG | Pheochromocytoma and Paraganglioma |
| PRAD | Prostate adenocarcinoma |
| READ | Rectum adenocarcinoma |
| SARC | Sarcoma |
| SKCM | Skin Cutaneous Melanoma |
| STAD | Stomach adenocarcinoma |
| TGCT | Testicular Germ Cell Tumors |
| THCA | Thyroid carcinoma |
| THYM | Thymoma |
| UCEC | Uterine Corpus Endometrial Carcinoma |
| UCS | Uterine Carcinosarcoma |
| UVM | Uveal Melanoma |
